# Antioxidant and Photoprotection Networking in the Coastal Diatom *Skeletonema marinoi*

**DOI:** 10.3390/antiox8060154

**Published:** 2019-06-01

**Authors:** Arianna Smerilli, Sergio Balzano, Maira Maselli, Martina Blasio, Ida Orefice, Christian Galasso, Clementina Sansone, Christophe Brunet

**Affiliations:** 1 Stazione Zoologica Anton Dohrn, Istituto Nazionale di Biologia, Ecologia e Biotecnologie marine, Villa Comunale, 80121 Napoli, Italy; arianna.smerilli@gmail.com (A.S.); sergio.balzano@szn.it (S.B.); maira.maselli@bio.ku.dk (M.M.); martina.blasio@szn.it (M.B.); ida.orefice@szn.it (I.O.); christian.galasso@szn.it (C.G.); christophe.brunet@szn.it (C.B.); 2 Department of Biology, University of Copenhagen, Strandpromenaden 5, 3000 Helsingør, Denmark

**Keywords:** light, ascorbic acid, phenolic compounds, flavonoids, photoprotection

## Abstract

Little is known on the antioxidant activity modulation in microalgae, even less in diatoms. Antioxidant molecule concentrations and their modulation in microalgae has received little attention and the interconnection between light, photosynthesis, photoprotection, and antioxidant network in microalgae is still unclear. To fill this gap, we selected light as external forcing to drive physiological regulation and acclimation in the costal diatom *Skeletonema marinoi*. We investigated the role of light regime on the concentration of ascorbic acid, phenolic compounds and among them flavonoids and their connection with photoprotective mechanisms. We compared three high light conditions, differing in either light intensity or wave distribution, with two low light conditions, differing in photoperiod, and a prolonged darkness. The change in light distribution, from sinusoidal to square wave distribution was also investigated. Results revealed a strong link between photoprotection, mainly relied on xanthophyll cycle operation, and the antioxidant molecules and activity modulation. This study paves the way for further investigation on the antioxidant capacity of diatoms, which resulted to be strongly forced by light conditions, also in the view of their potential utilization in nutraceuticals or new functional cosmetic products.

## 1. Introduction

Aerobic organisms need to deal with reactive oxygen species (ROS) which are harmful to their metabolism since high ROS concentrations can damage cellular machinery ultimately threatening cell survival; simultaneously ROS play also a role as secondary messengers. In all cells, mitochondria, NADPH oxidase (NOX) complexes and the enzyme lipoxygenase are major ROS sources. Photosynthetic eukaryotic cells possess, in addition, the chloroplasts, in which ROS are formed via energy transfer from chlorophyll or via electron transfer. Indeed, ROS intracellular concentration controls the photosystem II (PSII) activity and therefore photosynthesis and defense strategies [1]. The balance between toxicity, when ROS are in excess, and the signaling action requires cells to finely tune the ROS concentration [2,3,4], thanks to an efficient intracellular network composed by antioxidant molecules and enzymes. Antioxidants include molecules such as ascorbic acid (AsA), carotenoids, glutathione (GSH), tocopherols as well as phenolic compounds. AsA is a strong antioxidant component of the cell plasma [5]. AsA is also substrate of antioxidant enzymes such as peroxidases and violaxanthin de-epoxidase, thus contributing to dissipate excess energy [6]. Carotenoids occur in the chloroplast membranes interacting directly where photosynthesis-derived ROS are generated. Besides their role as photosynthetic pigments, carotenoids can efficiently quench peroxides and singlet oxygen thus preventing the formation of ROS [5,7,8,9]. GSH buffers the redox equilibrium of the cell by undergoing oxidation or reduction reactions, according to the redox potential of the cell. Specifically, GSH can act as an electron donor inactivating free radicals. Moreover, GSH is also a cofactor of several antioxidant enzymes [10]. Tocopherols are only produced by photoautotrophic taxa, they are lipophilic and can use resonance energy transfer to scavenge singlet oxygen, thus protecting PSII [11]. Phenolic compounds are present in all plants and derive from the shikimate-phenylpropanoids-flavonoids pathways [12]. They include a wide range of molecules with several phenol structural units. The most important phenolic compounds are flavonoids, which can donate both electrons or hydrogen atoms directly to ROS [13]. A wide range of flavonoids are present in photosynthetic microorganisms [14]. A recent study highlighted the diversity of flavonoids in phytoplankton and found that ferulic acid and apigenin are the dominant flavonoids in both cyanobacteria and eukaryotic microalgae [14]. In contrast with higher plants, their distribution and functions in microalgae are not fully clear [14,15]. Antioxidant enzymes are located in various cell compartments and include catalase (CAT), superoxide dismutase (SOD), and several peroxidases. Ascorbate peroxidase (APX) and glutathione peroxidase (GPX) accept, as substrates, AsA and GSH, respectively, in order to detoxify ROS. Furthermore, three additional enzymes, the monodehydroascorbate reductase (MDHAR), the dehydroascorbate reductase (DHAR), and the glutathione reductase (GR) contribute to regenerate the antioxidant substrates.

With the exception of carotenoids [16,17,18], the knowledge on antioxidant molecules and enzymes from marine microalgae is still scarce. Few studies focused on the concentration and composition of antioxidants in marine microalgae [17,19,20,21,22,23,24], and even fewer on the mechanisms for antioxidant defense in these microorganisms [24]. Indeed, both the photoprotective and antioxidant network appeared strongly controlled by light spectral composition and intensity, resulting in a complex regulation system, which allows planktonic diatoms to survive in their highly fluctuating light environment they naturally inhabit [24].

While the photoprotective mechanisms have been investigated in diatoms [25,26,27,28,29], only few studies investigated the role of the antioxidant network as a second defense line able to reduce the light stress [30,31]. Being light a crucial ecological axis in ruling the metabolism of photosynthetic organisms, and thus modulating the growth, the objective of this study was to investigate the impact of light intensity, photoperiod, and wave light distribution on the cellular concentrations of antioxidant molecules such as AsA and flavonoids, and total phenolic content. This knowledge can thus to be exploited to improve microalgal culturing with a productivity-driven purpose. Indeed, marine microalgae are gaining increasing attention for ecofriendly production of new antioxidant compounds. The ultimate aim of this study is to clarify the role of microalgal antioxidants in modulating light stress.

## 2. Materials and Methods

### 2.1. Skeletonema marinoi and Culture Conditions

The experiments were conducted on the diatom *Skeletonema marinoi* Sarno and Zingone (CCMP 2092); we selected *S. marinoi* since it is a cosmopolitan centric diatom broadly used in aquaculture [32] that can be cultured in different media [33] under different conditions of light intensity [34] and salinity [35]. 

This strain was grown at 20 °C in 4.5 L glass tanks in autoclaved seawater, pre-filtered through a 0.7 µm GF/F glass-fiber filter under water movement using an aquarium wave maker pump (Sunsun, JVP-110, Sunsun manufacturer, Zhoushan, China). A modified f/2 medium, in which the concentrations of phosphate, dissolved silica, vitamins, and trace metals are twice compared to those typically present in f/2 [36], was used.

Cells were pre-acclimated to a sinusoidal light distribution with a midday peak of 150 µmol photons m^−2^ s^−1^ and with a photoperiod equal to 12:12 dark:light, following the results obtained by [24,34,37]. 

The white light was composed by Red:Green:Blue (RGB) with a ratio of 10:40:50 and provided by a custom-built LED illumination system (European patent registration number: EP13196793.7), allowing to modulate the spectral composition and light intensity [38]. The three colors were provided at wavelengths of 460 nm (±36 nm, blue), 530 nm (±50 nm, green) and 626 nm (±36 nm, red).

Light intensity (Photosynthetically Active Radiation, PAR) was measured inside each tank by using a laboratory PAR 4 π sensor (QSL 2101, Biospherical Instruments Inc., San Diego, CA, USA).

### 2.2. Experimental Strategy

**Figure 1:**
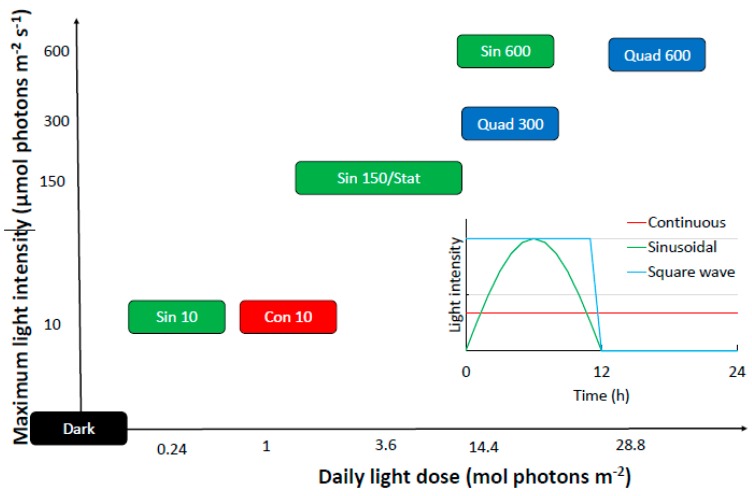
Details of the experimental setting used here. The different light wave distributions are explained in the inset. Treatments based on sinusoidal, quadratic or continuous light wave are shown in green, blue, and red, respectively. Refer to Table 1 for abbreviations.

All experiments were run in triplicate and consisted in monitoring the biological responses of *S. marinoi* after the shift from pre-acclimation light condition to different light conditions, spanning from prolonged darkness to very high light climate (Table 1). The light shift started after the 12 h dark period of the previous day. The experimental conditions are presented in the Figure 1 and described in Table 1.

The condition-oriented sampling strategy was carried out as follows. All the cultures were sampled at predawn (in the dark before the new condition operation), at 6 h (midday) and at 24 h (at the end of the dark period). Since phytoplankton cells are known to operate a rapid photoprotective mechanism when exposed to high (>200 μmol photons m^−2 ^s^−1^) light conditions [39] an additional sampling after 2 h from the light shift was performed in all the high light experiments (Sin 600, Quad 300 and Quad 600). Furthermore, since cultures incubated to square light wave experienced a sharp shift from 0 to 300 or 600 μmol photons m^−2 ^s^−1^, samples were harvested from these cultures also 10 and 30 min after the light shift in order to evaluate the short-time response of *S. marinoi* to cope with this unnatural and drastic enhancement of light.

### 2.3. Ancillary Data

#### 2.3.1. Cell Concentration and Growth Rate

To assess cell density, 2 mL of cell suspension were collected from each tank and fixed with Lugol’s iodine solution (1.5% *v*/*v*). Then, 1 mL of this solution was used to fill a Sedgewick Rafter counting cell chamber. Cells were then counted using a Zeiss Axioskop 2 Plus light microscope (Carl Zeiss, Göttingen, Germany).

The growth rate was estimated from cell concentration measurements using the following equation:

µ (d^−1^) = ln(C_n−1_/C_n_)/(t_n_ − t_n−1_),

where µ is the growth rate, C_n−1_ and C_n_ are cell concentrations (mL^−1^) at day n − 1 (t_n−1_) and day n (t_n_).

#### 2.3.2. Photochemical Efficiency of the Photosystem II and Non-Photochemical Quenching Measurements

To assess the photosynthetic capacities and the photophysiological state of phytoplankton cells, active chlorophyll *a* (Chl-*a*) fluorescence was measured using a DUAL-PAM fluorometer (Heinz Walz GmbH, Effeltrich, Germany). The photochemical efficiency of the PSII (F_v_/F_m_) was estimated by:

ϕ_p_= (F_m_ − F_0_)/F_m_ = F_v_/F_m_,

where F_v_ is the variable fluorescence (F_v_ = F_m_ − F_o_). 

The measurement of F_o_ was done using light of low intensity (1 μmol photons m^−2^ s^−1^) and low frequency (approximately 25 Hz). F_m_ was measured by applying a short and intense flash of actinic light which completely reduces QA. In our case, the saturation flash of bright red light (655 nm) were applied at an intensity of 2400 μmol photons m^−2^ s^−1^ for a duration of 450 ms. F_v_/F_m_ corresponds to the maximal photochemical efficiency of the PSII (or the maximal light utilization efficiency of PSII) and dark acclimation for 15 min allows the recovery of photosystems II, this leading to reliable measurements of F_o_ [38]. 

Estimation of the non-photochemical quenching is calculated by the Stern–Volmer expression [40]:

NPQ = (F_m_ − F’_m_)/F’_m_ = F_m_/F’_m_ − 1.

The estimation of NPQ consisted of measuring F_o_ and F_m_ on 15 min dark-acclimated samples and then measuring F_m_’ and F_0_′ every minute on the same sample illuminated by an actinic light (setup at 399 μmol photons m^−2^ s^−1^) for 10 min.

#### 2.3.3. Electron Transport Rate-Light Curves Determination

The electron transport rate (ETR) vs. irradiance (E) curves were determined on 15-min dark-acclimated samples by applying a series of 10 increasing intensity actinic lights (composed by 2/3 of blue and 1/3 of red light, lasting 1.5 min each, ranging from 1 to 1222 μmol photons m^−2^ s^−1^). The photochemical efficiency of the PSII was measured on the 15-min dark-acclimated sample, while the light utilization efficiency of the PSII (ΔΦ) was measured after each actinic light level.

The relative ETR, taking into account the part of incident light energy effectively absorbed by the photosystem, was calculated as follows:

relETR = F’_v_/F’_m_∙E∙0.5∙a *,

where E is irradiance, and *a* * is the cell specific absorption coefficient expressed in m^2^ cell^−1^ [24]. A factor of 0.5 was applied since it is assumed that half of the incident light is absorbed by the PSI and half by the PSII. The relative ETR is expressed in nmol e^−^ h^−1^ cell^−1^. 

Determination of relETR_max_ was retrieved according to the equation of Eilers and Peeters (1988) [41].

### 2.4. Pigment Analysis

Pigment analysis was conducted by High Performance Liquid Chromatography (HPLC) as described by Smerilli et al. [24]. An aliquot of algal culture (10 mL) was filtered on GF/F glass-fiber filter (25 mm, Whatman, Maidstone, UK) and immediately stored in liquid nitrogen until further analysis. Pigments were extracted by mechanical grounding for 3 min in 2 mL of absolute methanol. The homogenate was then filtered onto Whatman 25-mm GF/F filters and the volume of the extract accurately measured. Prior to injection into the HPLC, 250 μL of 1 M ammonium acetate were added to 500 μL of the pigment extract and incubated for 5 min in darkness at 4 °C. This extract was then injected in the 50 μL loop of the Hewlett Packard series 1100 HPLC (Hewlett-Packard, Wilmington, NC, USA). The reversed-phase column (2.6 mm diameter C8 Kinetex column; 50 × 4.6 mm; Phenomenex^®^, Torrance, CA, USA) corresponded to an apolar stationary phase composed of silica beads possessing aliphatic chains of eight carbon atoms (C8). The temperature of the column was steadily maintained at 20 °C and the flow rate of the mobile phase was set up at 1.7 mL min^−1^. 

The mobile phase was composed of two solvents mixtures: A, methanol:0.5 N aqueous ammonium acetate (70:30, *v*/*v*) and B, absolute methanol. During the 12-min elution, the gradient between the solvents was programmed: 75% A (0 min), 50% A (1 min), 0% A (8 min), 0% A (11 min), 75% A (12 min). 

Pigments were detected at 440 nm using a Hewlett Packard photodiode array detector model DAD series 1100 which gives the 400–700 nm spectrum for each detected pigment. A fluorometer (Hewlett Packard standard FLD cell series 1100) with excitation at 410 nm and emission at 665 nm allowed the detection of fluorescent molecules (chlorophylls and their degraded products). Pigments were identified based on their retention time and quantified based on pure standards from the D.H.I. Water and Environment (Hørsholm, Denmark) as described previously [38]. 

### 2.5. Antioxidant Molecules and Antioxidant Activity Analysis 

#### 2.5.1. Ascorbic Acid Content Determination

To assess the ascorbic acid (AsA) content in cells, the procedure modified from [42] was the following. A 150 mL volume of culture was centrifuged at 3600 g for 15 min at 4 °C (DR15P centrifuge, B. Braun Biotech International, Melsungen, Germany), the pellet was weighed, flash frozen in liquid nitrogen, and stored at −20 °C. Pellets were resuspended in 5% trichloroacetic acid (TCA) and sonicated for 1 min with a microtip at 20% output on ice (S-250A Branson Ultrasonic). Cell debris were precipitated by centrifugation at 5000× *g* for 5 min at 4 °C. The supernatant was then used for spectrophotometric analysis after mixing it with a reagent. The reagent consisted of a 0.5% solution of 2,2′-dipyridyl mixed with an 8.3 mM ferric ammonium sulfate solution in 15% (*v*/*v*) o-phosphoric acid in a ratio 4 to 1. Supernatant and reagent were mixed (1:5) immediately before use. After 1 h the absorbance was read at 520 nm and AsA concentration was calculated thanks to factor calibration retrieved from calibration curves using AsA standards. AsA concentration is reported in fg AsA cell^−1^.

#### 2.5.2. Preparation of the Methanolic Extracts

For the determination of 2,2′-azino-bis (3-ethylbenzothiazoline-6-sulphonic acid) (ABTS) radical scavenging activity, total phenolic content (TPC) and total flavonoid content (TFC), the pellets were prepared as follows. Cells were re-suspended in methanol and sonicated for 1 min with a microtip at 20% output on ice (S-250A Branson Ultrasonic). The suspension was left for 30 min at room temperature in the dark, and then was centrifuged at 3600× *g* for 10 min at 4 °C. Supernatant was collected and the pellet was re-suspended in an equal volume of methanol and left other 30 min at room temperature in the dark. The suspension was centrifuged again in the same conditions, and the two supernatants were combined. 

#### 2.5.3. Total Phenolic Content

Polyphenols in plant extracts react with specific redox reagents (Folin-Ciocalteu reagent) to form a blue complex that can be quantified by visible-light spectrophotometry. Total phenolic content (TPC) was estimated by the Folin–Ciocalteu method [43] as described by Li and collaborators [44].

Briefly, 200 µL of the sample was mixed with 1 mL of Folin-Ciocalteu’s phenol reagent, pre-diluted in distilled water 1:10 *v*/*v*. After 4 min, 800 µL 75 g/L Na_2_CO_3_ were added to the mixture, shacked vigorously and stored at room temperature for 2 h. The absorbance was read at 765 nm. Gallic acid was used for the standard calibration curve. The results were expressed in fg of gallic acid equivalents (GAEq) cell^−1^.

#### 2.5.4. Total Flavonoid Content

The total flavonoid content was estimated by aluminum chloride colorimetric method [45]. Briefly, 600 µL of sample were pre-diluted 1:2 *v*/*v* in methanol 80% *v*/*v* and mixed with an equal volume of AlCl_3_ 2%. The mix was shaken and incubated at room temperature for 1 h. The absorbance was measured at 410 nm and quercetin was used for the standard calibration curve. The results were expressed in fg of quercetin equivalents (QEq) cell^−1^.

#### 2.5.5. ABTS Radical Scavenging Activity

The antioxidant activity was assessed by the 2,2′-azino-bis (3-ethylbenzothiazoline-6-sulphonic acid) (ABTS) radical scavenging activity assay. The scavenging activity of ABTS radical was measured following [16]. The ABTS free radical was generated by mixing 7 mM ABTS diammonium salt with 2.45 mM potassium persulfate and stored overnight at room temperature. The solution was diluted with methanol till the absorbance at 734 nm reached 0.70 ± 0.01 units. Then one part of sample was mixed with three parts of ABTS radical solution. The mix was shaken and left 1 h at room temperature in the dark. The absorbance was read at 734 nm. Ascorbic acid was used for the standard calibration curve. The results were expressed in fg of ascorbic acid equivalents (AEq) cell^−1^.

### 2.6. Statistical Analysis

Calculations of mean, standard deviation, variance, coefficient of variation (CV), Student’s *t*-test for mean comparison, Spearman rank correlation, analysis of variance (ANOVA) and Tukey test for multiple comparisons were performed using the PAST software package, version 3.10 [46]. 

## 3. Results

### 3.1. Growth Rate and Photosynthesis

After 24 h from the light shifts, in all the experimental conditions the growth rate of *S. marinoi* decreased (Table 2), in contrast with what observed in the control condition, revealing a physiological stress induced by the variations of light environment. The highest decrease in cell abundance was observed for cultures shifted to square wave light distribution at 600 μmol photons m^−2^ s^−1^ and to continuous light (Table 2).

Photosynthetic electron transport rate (ETR) did not vary significantly over time under Sin 150, dark conditions, and Sin 10. Significant increases were observed under continuous light (Con 10), stationary conditions (Sin 150 Stat), and for cultures incubated under high light conditions (Table 3). The photochemical efficiency of photosystem II (Fv/Fm, Table 3) decreased significantly after 6 h for cultures exposed to square-wave light distribution type. Fv/Fm restored after 24 h in Quad 600, whereas it did not reach its initial value in Quad 300 probably because of photoinhibition. Fv/Fm decreased over time in Sin 10, Con 10 and Sin 600 treatments, whereas it did not change significantly for the control and the dark treatments. 

### 3.2. Photosynthetic Pigments

The cellular concentration of Chl-*a* varied differently depending on the light climate cells were exposed to. Decreases were observed in the Sin 150 Stat (6 h), whereas sharp increases occurred in the dark treatment (6 h) and in the Con 10 (24 h). In the Quad 300 the concentration of Chl-*a* decreased (24 ± 1 to 15.1 ± 6.0 fg cell^−1^) after 2 h and then increased again in the following 4 h (22.2 ± 2.1 fg cell^−1^). The cellular concentration of fucoxanthin (fuco) increased in the cultures incubated under low light conditions (Con 10 and Sin 10) and did not change significantly in the other cultures except in Quad 300, where it decreased after 2 h and then increased again during the following 22 h (Table 3). Under control condition (Sin 150), the increase in Chl-*a* content per cell at midday was attributed to cell cycle progression, and its decrease at 24 h was the result of cell division occurring during night (Table 3). Conversely, under Sin 600 or Quad 600 and 300, cell pigment content did not change significantly with time, probably because of the arrest of cell cycle progression or related to the two antagonist effects of high light regulation (lowering Chl-*a* concentration) and cell cycle progression (increasing it). 

Prolonged darkness induced an increase of Chl-*a* with time revealing an acclimation strategy in the reaction centers of photosystems (Table 3). Under Sin 10, after 6 h Chl-*a* decreased significantly while Fuco tended to increase.

Under Con 10, both Fuco and Chl-*a* were strongly enhanced the second day of experiment (Table 3). 

### 3.3. Photoprotection: NPQ and Xanthophyll Cycle

**Figure 2:**
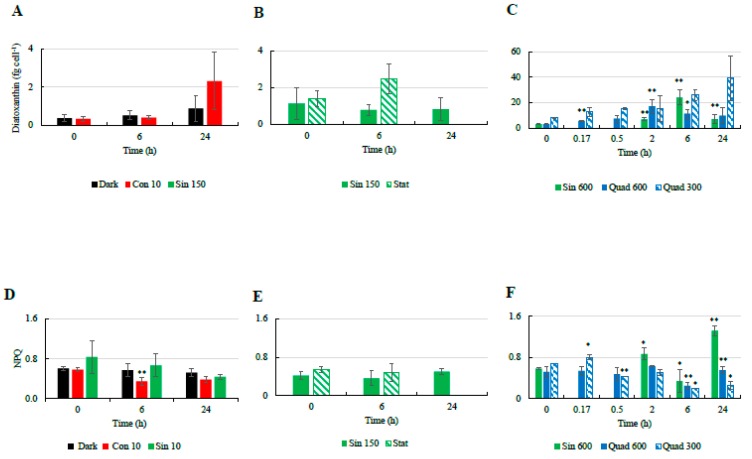
Temporal changes in the cellular concentrations of diatoxanthin ((**A**): low light climates, (**B**): moderate light, (**C**): high light climates) and non-photochemical quenching (NPQ; (**D**): low light climates, (**E**): moderate light, (**F**): high light climates) in the different culturing treatments of *Skeletonema marinoi* CCMP 2092. Refer to Table 1 for abbreviations. “*” means significantly different from time 0 (*p* < 0.05); “**” means significantly different from time 0 (p < 0.01).

Major changes in NPQ as well as the pigments related to the xanthophyll cycle were mostly observed in the cultures exposed to high light. Indeed, the NPQ was found to be more variable in cultures incubated under Sin 600 and square light wave conditions compared to the other treatments (Figure 2). Under continuous light (Con 10) the cellular concentration of Dt increased and the NPQ decreased over time. While the increase in Dt (Figure 2A–C) mostly occurred in the last 18 h, the NPQ decreased during the first 6 h (Figure 2D–F). Cells incubated under sinusoidal conditions in both exponential (Sin 150) and stationary (Sin 150 stat) phases did not exhibit significant infradiel variations in Dt and NPQ (Figure 2B,D). Under Sin 600, NPQ increased rapidly in the first 2 h (*p* < 0.05), coming back to the pre-dawn values at midday (Figure 2F). At 24 h NPQ was significantly higher (*p* < 0.001) with respect to the previous predawn day value (Figure 2F). Under Quad 300, NPQ slightly increased after 10 min and later on decreased progressively reaching the lowest value after 6 h (*p* < 0.05, Figure 2C). Conversely, Dt progressively increased reaching its maximal concentration at 6 h (*p* < 0.05, Figure 2C) and the DES significantly increased after 30 min (data not shown). Under Quad 600, the cellular concentration of Dt increased after 2 h and then decreased again; the NPQ was fairly constant within the first 2 h while it decreased significantly after 6 h (*p* < 0.05, Figure 2F).

**Figure 3:**
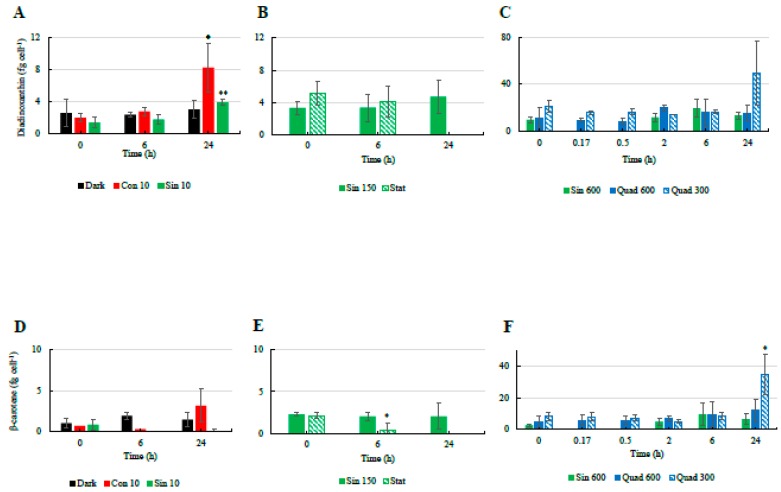
Temporal changes in the cellular concentrations of diadinoxanthin ((**A**): low light climates, (**B**): moderate light, (**C**): high light climates) and β-carotene ((**D**): low light climates, (**E**): moderate light, (**F**): high light climates)in the different culturing treatments of *S. marinoi* CCMP 2092. Refer to Table 1 for abbreviations. “*” means significantly different from time 0 (*p* < 0.05); “**” means significantly different from time 0 (p < 0.01).

The cellular concentrations of Dd and β-car did not change significantly over time in most cases (Figure 3). A small increase in both Dd and β-car was only observed in the Con 10 treatment after 24 h since the beginning of the experiments (Figure 3A,D). NPQ and the concentration of the photoprotective pigment Dt were thus not correlated (Table 4), as previously observed under sinusoidal light distribution [24,47]. 

### 3.4. Antioxidant Molecules and Activity 

**Figure 4:**
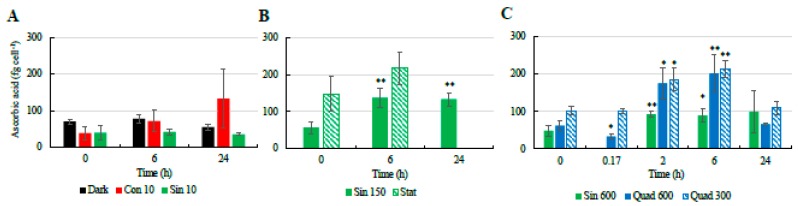
Temporal changes in the cellular concentrations of ascorbic acid ((**A**): low light climates, (**B**): moderate light, (**C**): high light climates) in the different culturing treatments of *S. marinoi* CCMP 2002. Refer to Table 1 for abbreviations. “*” means significantly different from time 0 (*p* < 0.05); “**” means significantly different from time 0 (p < 0.01).

**Figure 5:**
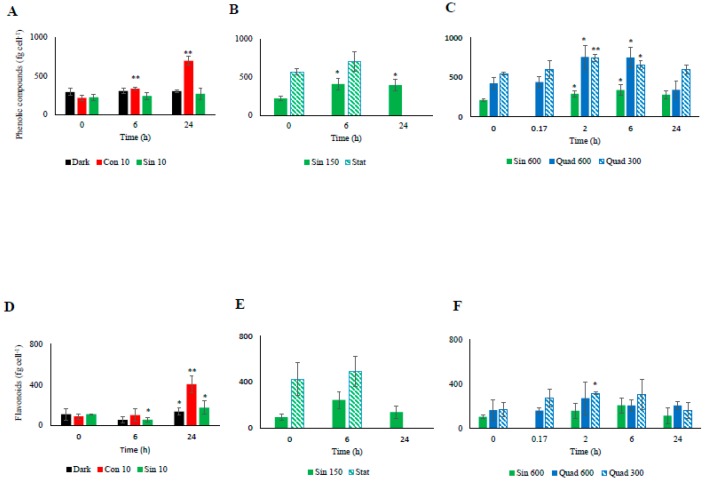
Temporal changes in the cellular concentrations of phenolic compounds ((**A**): low light climates, (**B**): moderate light, (**C**): high light climates) and flavonoids ((**D**): low light climates, (**E**): moderate light, (**F**): high light climates) in the different culturing treatments of *S. marinoi* CCMP 2002. Refer to Table 1 for abbreviations. “*” means significantly different from time 0 (*p* < 0.05); “**” means significantly different from time 0 (p < 0.01).

Similarly to the NPQ and the xanthophyll-cycle related pigments, most changes in the cellular concentrations of antioxidant molecules occurred in the cultures incubated under high light (Figure 4 and Figure 5). Under the control condition (Sin 150), the cellular concentration of ascorbic acid (AsA) increased at midday (Figure 4). The phenolic content followed the same trend, with higher values found at midday compared to pre-dawn (Figure 5A–C). As AsA and phenolic content, flavonoids increased at midday compared to pre-dawn (Figure 5D–F). In contrast when cells entered stationary phase, these daily variations disappeared and the flavonoids, phenolics, and AsA concentrations stabilized on higher values with respect of those recorded in the exponential phase (Figure 4 and Figure 5).

**Figure 6:**
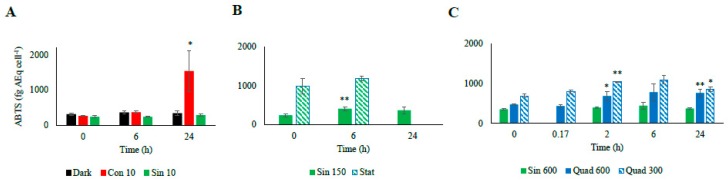
Radical scavenging activity of the different culturing treatment based on the assay of 2,2′-azino-bis (3 ethylbenzthiazoline-6-sulfonic acid, ABTS; (**A**): low light climates, (**B**): moderate light, (**C**): high light climates). Data are indicated in fg of ascorbic acid equivalent per cell (fg AEq cell^−1^). Refer to Table 1 for abbreviations. “*” means significantly different from time 0 (*p* < 0.05); “**” means significantly different from time 0 (p < 0.01).

Consistent with this observation, the ABTS test reflected the trend observed for the antioxidant molecules, following infradiel variations, with an enhancement at midday, and further increase during the stationary phase (Figure 6).

Antioxidant activity (ABTS) and antioxidant compounds (AsA, phenolics, and flavonoids content) were significantly correlated with all the parameters related to photoprotection (Dt and its two precursors Dd and β-car) except NPQ as well as light intensity (Table 4). The situation changed a little when cells entered into the stationary phase, in which light was no longer the only parameter inducing antioxidant response (lack of correlation, Table 4). ABTS activity was linked to phenolic content, AsA, and Dt, these three parameters being linked between them (Table 4). In this condition, a negative correlation between Dt and β-car was noticed on the opposite to the exponentially grown cells, revealing that Dt might be enhanced from β-car pool that was not fully replenished.

Under Sin 600, AsA conserved the infradiel variation previously observed (Figure 4C). AsA concentration doubled already at 2 h, keeping its concentration fairly constant towards the end of the experiments. The total phenolic content and flavonoids concentration followed a trend similar to AsA in Sin 600, although the increase was observed after 6 h since the beginning of the experiments (Figure 5C,F). Under Quad 600 the infradiel trend of AsA previously observed was still present (Figure 4C). Intriguingly, AsA concentration was halved in 10 min revealing its very fast consumption, then AsA was recycled and newly synthesized, reaching the highest values at 2 and 6 h (*p* < 0.05 and *p* < 0.01, respectively, Figure 4C). The increase between the time 0 and 2–6 h was greater than the increase observed in the control condition, being of circa three times against 2.4 in Sin 150 and 1.8 in Sin 600 (Figure 4B,C).

The ABTS test paralleled the increase of the antioxidants’ concentration, remaining high at 6 h (Figure 6). After one day since the light shift, the antioxidant capacity was still higher than T_0_ (*p* < 0.01), while the antioxidant molecules decreased or were stable with respect to the previous day (Figure 4, Figure 5 and Figure 6). 

Under both square light wave conditions AsA content (Figure 4C) did not vary within the first minutes of the experiment, increasing at 2 and 6 h (*p* < 0.05) and restoring the starting values after 24 h. The increase in AsA found in Sin 600 was lower than the one observed in Quad 300 and Quad 600 (Figure 4C). The phenolic content in both Quad 300 and Quad 600 peaked at 2 h (*p* < 0.05), then decreased restoring the initial values at 24 h while flavonoids did not show any significant variation (Figure 5C,F). The ABTS followed the same trend of AsA, increasing at 2 h and keeping high values at 6 h (*p* < 0.05, Figure 6).

During the shift to Sin 600, the ABTS activity was correlated with light distribution as well as with phenolic content and the pigments Dt and β-car (Table 5). On the difference to the Sin 150 condition, AsA and flavonoids seemed to do not be involved in ABTS activity (lack of correlation, Table 5), conversely to what was observed in Sin 150. When cells coped with high light square wave distribution, the correlations changed again. Under Quad 600, ABTS activity was only related to flavonoids content (Table 5) while phenolic content was linked to Dt. Under Quad 300, i.e., a square wave distribution with a daily light dose similar to Sin 600, the ABTS activity was related to AsA, with the latter negatively correlated to NPQ (Table 5).

Under prolonged darkness, phenolic compounds, flavonoids, AsA, and ABTS did not vary significantly over time (Figure 4, Figure 5 and Figure 6). Under continuous light the cellular concentration of AsA did not change over time while that of both phenolic compounds and flavonoids doubled and quadrupled, respectively, after 24 h (Figure 4). The increase of ABTS by five fold after 24 h in the Con 10 treatment was coupled to the antioxidant molecules increase (Figure 5 and Figure 6).

Changes in the cellular concentration of antioxidants, as well as ABTS over time were not significant for both Sin 10 and dark treatments (Figure 5A–D and Figure 6A). In continuous dark, ABTS scavenging activity was significantly related to the Dt and β-car while none of the antioxidant molecules was correlated to ABTS (Table 6). Conversely, under Con 10, the unique parameter significantly involved in the ABTS activity was the flavonoids concentration. Under Sin 10, ABTS activity was correlated to phenolic compounds (but not flavonoids, Table 6). These results highlighted the diversity of the responses of the cells when copying with different low light treatments. 

## 4. Discussion

Current results highlighted that *Skeletonema marinoi* turned out to be rich in phenolic compounds, which are the most widespread antioxidant substances in photosynthetic organisms [48,49]. Phenolic compounds are able to act directly against radical species as well as indirectly via the inhibition of pro-oxidant enzymes such as lipoxygenase or through metal chelation, preventing the occurrence of the Haber–Weiss and the Fenton reactions, which are important sources of radical species [50]. Although some studies reported that phenolic compounds are the main contributors to microalgal antioxidant capacity [17,24,48,51], the microalgal phenolic content is studied little [14,17,20]. The most abundant phenolic compounds in phytoplankton are phloroglucinol, *p*-coumaric acid as well as flavonoids such as ferulic acid and apigenin [14]. Among them, few studies explored their modulation in response to environmental forcing changes [44,49,52,53]. As reported previously [17,24], the content of phenolic compounds in microalgae is higher than in macroalgae and many higher plants. Assuming a dry weight per cell equivalent to 55 pg as in *Skeletonema costatum* [54], we estimate an average phenolic content ≈ 5.5 mg GAE g^−1^ DW, with values up to 12.7 mg GAE g^−1^ DW in some conditions. These values are in the range of previous estimations on the same species object of the present study [24] and in the higher range of results from different studies [17,44,48,51]. Yet, another study [20] reported high phenolic content (8–17.5 mg GAE g^−1^ DW) in four microalgae from different taxa: *Nannochloropsis oceanica* (Eustigmatophyceae), *Chaetoceros calcitrans* (diatom), *Skeletonema costatum* (diatom), and *Chroococcus turgidus* (cyanophyte). 

Among the phenolic compounds family, recent findings demonstrated diatoms’ ability to produce flavonoids [49], which display relevant antioxidant activity and act as signaling molecules able to up-regulate the defense strategies [13,49]. In most of the light conditions tested in our study, flavonoids’ concentration generally shows the same trend observed for the phenolic compounds. Flavonoids are located in different organelles, including chloroplasts where they play a key photoprotective role [55,56,57]; in particular, they can scavenge radical species and stabilize membranes containing non-bilayer lipids, such as monogalactosyldiacylglycerol (MGDG) [58]. 

Our study shows that flavonoids are strictly related to ABTS scavenging activity under unnatural light stress, such as continuous (0:24 h, light: dark) and very high light (600 μmol photons m^−2^ s^−1^; Quad 600), conversely to AsA. This might confirm the powerful capacity of flavonoids to act as defense against stress as photoprotector [59]. Their concentration ranges from circa 50 to 400 fg quercetin equivalent (QEq) cell^−1^, corresponding to ≈ 1 to 8 mg quercetin equivalent (QEq) g^−1^ DW. Interestingly, these values correspond to concentrations reported in a wide range of vegetables, fruits or higher plants [60,61,62].

AsA concentration in *S. marinoi* is also high, with values spanning from 10 to 300 fg AsA cell^−1^ (≈1.8–5.5 mg AsA g^−1^ DW) in the range of the values previously reported for the same species [24], as well as other phytoplankters [32,63,64]. The latter study highlighted the high variability of AsA concentration among different groups and between exponential and stationary growth phases, with concentrations up to 16 mg AsA g^−1^ DW. Our results highlight the huge potential of *S. marinoi*, the diatom model used in this study, as alternative source of antioxidant molecules. This study also shows the relevance of light driven-modulation on the intracellular concentrations of these molecules.

Current results highlight a substantial infradiel variability in the cellular concentrations of antioxidants. The increase in antioxidants observed at midday confirms the role of light in controlling antioxidant synthesis; antioxidants counteract the detrimental effect of the ROS which are produced as consequence of light exposure, as already observed in higher plants [65,66]. In the absence of light or under an extremely low sinusoidal light, infradiel variations of protective or antioxidant responses disappear, highlighting a direct light stimulus control, excluding an internal circadian clock, of these variations. A circadian clock synchronized with predictable daily environmental cyclic variations generally represents an evolutionary adaptation able to increase the fitness of the organism [67]. Instead, under the highly fluctuating light environment naturally experienced by diatoms, which frequently move along the water column, the presence of a rigid scheme ruling cell physiology could be a disadvantage. A better strategy might consist of promptly modifying the metabolism following the external stimuli, resulting in a great plasticity, which is a known feature attributed to diatoms. 

In contrast with what was reported under sinusoidal light distribution, square wave distribution does not induce cyclical infradiel variability. The sinusoidal high light distribution, although slowing microalgal growth, is well tolerated, thanks to the activation and functioning of the antioxidant-photoprotective network. By contrast, square wave distribution with high light intensity strongly affects cell performance impairing the normal functioning of the defense processes network. 

Light climate changes experienced by cells induce an uncoupling of the regulative responses (photoprotection vs. AsA, phenol and flavonoid contents) compared to the synergy of these photoresponses observed under pre-acclimation light (Sin 150). Sinusoidal high light exposition leads ABTS scavenging activity to be related to phenolic content as well as Dt and β-car while a non-significant role of flavonoids or AsA content is observed. Parallel responses of Dt and phenolic compounds’ concentrations have been already reported [24], along with no significant relationship between Dt and NPQ ([24,37,47]; this study) confirming an alternative role of this pigment, which is likely to have an additional antioxidant function. Under sinusoidal light distribution, with either moderate or high intensity, significant contribution of Dt in ROS scavenging activity is detected. Intriguingly, the relationship between Dt and ABTS is always accompanied by the significant correlation between β-car and ABTS (except when cells enter the stationary phase) that might reveal a similar role of these two pigments in ROS scavenging. The discrepancy between NPQ and Dt is related to an earlier NPQ response compared to Dt as observed under Sin 600, with the highest NPQ recorded after 2 h and subsequently decreasing. This uncoupling between NPQ and Dt confirms the role of NPQ as first defense strategy against light-related stress and that of Dt as a less quick ROS quencher [47]. 

In Quad 600 the significant role of flavonoids into the ABTS scavenging activity, by contrast to the other phenolic compounds, agree with the fact that flavonoids are known to have strong antioxidant activity [68,69,70], together with a relevant role in photoprotection [58] that relies on their enhanced concentration in chloroplasts, sites of light-driven ROS production [71,72]. 

The peculiar response of AsA under Quad 600, with a decrease recorded after 10 min of light exposure, might be due to its fast consumption to counteract the oxidative process induced by abrupt and strong high light exposure. 

By contrast in lower light square wave distribution (Quad 300) AsA seems to control ABTS scavenging activity since they are both significantly correlated.

Under low light conditions, different bioactive compounds families with respect to the light climate modulate ABTS scavenging activity.

Under prolonged darkness the increased concentration of Dt is induced by the chlororespiration-dependent trans-thylakoid ΔpH [39,73,74], and significantly linked to ABTS scavenging activity. Under very low light conditions (Sin 10), ABTS only significantly relies on phenolic content, as it was also observed—together with Dt—in Sin 600. This very low light intensity does not determine any increase in Dt, probably because of the absence of chlororespiratory pathway development as observed in prolonged darkness. 

By contrast, the continuous low light causes a strong impairment of the normal cell functioning inducing high cell mortality. Under this condition, such as under Quad 600 ABTS scavenging activity is only related to flavonoids content.

Not only light distribution and/or intensity, but also culture age changes dramatically the photoresponses of the cells. All the antioxidant molecules as well as Dt increase during cell senescence. The accumulation AsA has been already observed in the senescent diatom *S. marinoi* [64]. The infradiel variations observed during the active growth phase were disrupted during the stationary phase, vouching for the drastic changes to which the cells were subjected [75]. Conversely to exponentially grown cells, NPQ remains high at midday together with the antioxidant capacity and molecule concentration. The integrated defense strategy development suggests the high level of ROS produced in senescent cultures. In higher plants, the early event of cell senescence is the inactivation of the enzyme Rubisco [76,77] not paralleled by a loss of the thylakoid proteins, which happens at a later time [77]. Therefore, the potential exposure to increasing light induces the development of the first defense mechanism represented by NPQ and, subsequently, the antioxidant network is involved in the scavenging of the ROS, which are produced by the accumulation of electrons from the photosynthetic process.

## 5. Conclusions

In conclusion, phenols do account for scavenging activity in the case of natural gradual light variations (moderate, high, or extremely low light), while flavonoids are the family of compounds “selected” in the case of un-natural and very stressful change of light (Con 10, Quad 600).

This study provides evidence of the interconnection between xanthophyll-cycle-relied photoprotection and synthesis of antioxidant molecules. This study highlights the great potential of diatoms as alternative source of natural antioxidant molecules such as carotenoids, phenolic compounds, flavonoids, and ascorbic acid—as well as on the role of light manipulation as an effective tool for enhancing antioxidant molecules synthesis in diatoms.

## Figures and Tables

**Table 1:** Experimental strategy.

Condition	Growth Phase at T_0_	Photoperiod (Light:Dark)	Light Intensity (μmol Photons m^−2^ s^−1^)	Daily Light Dose (mol Photons m^−2^ d^−1^)	Light Distribution
Sin150	Exponential	12:12	150	3.6	Sinusoidal
Stat	Stationary	12:12	150	3.6	Sinusoidal
Dark	Exponential	00:24	0	0	-
Sin10	Exponential	12:12	10	0.24	Sinusoidal
Con10	Exponential	24:00	10	1	Continuous
Sin600	Exponential	12:12	600	14.4	Sinusoidal
Quad300	Exponential	12:12	300	14.4	Square wave
Quad600	Exponential	12:12	600	28.8	Square wave

**Table 2:** Cell concentration and growth rate at time T_o_ and after 24 h from the start of the experiment.

Condition	Cell Concentration (10 ^5^ Cells mL^−1^)		Growth Rate (µ, d^−1^)	
	0 h	24 h	0 h	24 h
Sin150	2.9 ± 0.4	6.7 ± 0.5	1.2 ± 0.1	1.2 ± 0.3
Stat	5.3 ± 0.7	4.0 ± 0.4	−0.4 ± 0.3	-
Dark	4.6 ± 0.5	4.0 ± 0.4	1.0 ± 0.2	−0.1 ± 0.1
Sin10	4.4 ± 0.6	4.8 ± 0.4	1.0 ± 0.2	0.1 ± 0.1
Con10	6.8 ± 0.5	0.8 ± 0.4	1.0 ± 0.1	−1.5 ± 0.6
Sin600	7.2 ± 0.4	8.1 ± 2.1	1.1 ± 0.1	0.1 ± 0.2
Quad300	4.7 ± 1.0	1.8 ± 2.0	0.5 ± 0.2	−1.5 ± 1.4
Quad600	5.9 ± 1.7	1.5 ± 1.0	0.8 ± 0.2	−1.5 ± 0.4

**Table 3:** Electron photosynthetic rate (ETR, nmol e^−^ h^−1^ cell^−1^), Fv/Fm, Chlorophyll-*a* concentration (Chl-*a,* fg cell^−1^), and Fucoxanthin concentration (Fuco, fg cell^−1^) measured under the different light conditions.

	Sampling Time (h)	Sin150	Dark	Sin10	Con10	Sin600	Quad300	Quad600
ETR	0	22.6 ± 6.2	11.55 ± 3.01	23.21 ± 5.70	82.01 ± 26.81	15.92 ± 2.28	30.14 ± 14.60	20.56 ± 5.31
	0.17							
	0.5						33.60 ± 5.20	19.79 ± 1.00
	2					26.18 ± 6.25	42.03 ± 15.53	21.39 ± 1.00
	6	25.1 ± 3.7	8.58 ± 1.68	23.82 ± 4.63	47.85 ± 7.46	16.20 ± 3.03	26.71 ± 0.72	26.72 ± 1.02
	24	19.2 ± 7.9	8.33 ± 3.74	15.39 ± 3.52	22.56 ± 3.03	23.92 ± 3.97	32.87 ± 0.23	35.35 ± 1.00
Fv/Fm	0	0.80 ± 0.06	0.82 ± 0.08	0.97 ± 0.04	0.92 ± 0.04	0.78 ± 0.03	0.78 ± 0.00	0.79 ± 0.03
	0.17						0.82 ± 0.11	0.81 ± 0.08
	0.5						0.81 ± 0.06	0.81 ± 0.10
	2					0.77 ± 0.06	0.82 ± 0.05	0.82 ± 0.04
	6	0.79 ± 0.04	0.94 ± 0.04	0.93 ± 0.08	0.79 ± 0.04	0.74 ± 0.08	0.56 ± 0.03	0.65 ± 0.05
	24	0.74 ± 0.01	0.72 ± 0.09	0.78 ± 0.05	0.68 ± 0.05	0.88 ± 0.01	0.63 ± 0.00	0.75 ± 0.01
Chl-*a*	0	93.7 ± 13.9	49.50 ± 1.35	55.70 ± 2.74	23.80 ± 1.30	87.50 ± 2.40	237.80 ± 8.40	10.39 ± 4.41
	0.17						242.70 ± 3.96	17.40 ± 3.73
	0.5						242.70 ± 5.30	20.07 ± 8.53
	2					96.50 ± 1.99	150.60 ± 6.04	17.30 ± 7.37
	6	126.5 ± 22.0	80.70 ± 1.13	27.00 ± 0.98	17.40 ± 0.77	104.00 ± 3.16	221.70 ± 2.30	20.67 ± 2.14
	24	102.00 ± 5.74	78.30 ± 2.16	21.50 ± 2.53	22.80 ± 11.80	98.30 ± 3.74	636.40 ± 61.30	26.75 ± 8.94
Fuco	0	32.0 ± 5.0	22.5 ± 10.7	18.1 ± 8.8	20.2 ± 4.7	39.3 ± 10.0	95.10 ± 18.00	44.2 ± 12.0
	0.17						97.9 ± 8.4	64.3 ± 6.5
	0.5						109.9 ± 5.7	66.7 ± 26.8
	2					35.4 ± 7.6	60.6 ± 16.0	78.8 ± 25.3
	6	26.4 ± 10.2	27.60 ± 0.70	28.1 ± 9.2	36.4 ± 1.2	33.1 ± 5.3	79.6 ± 16.0	57.7 ± 16.3
	24	35.6 ± 15.0	28.6 ± 11.0	52.3 ± 8.4	101.60 ± 2.58	37.2 ± 9.8	287.60 ± 29.93	89.5 ± 59.0

**Table 4:** Spearman correlation matrix between the antioxidant capacity, antioxidant molecules, and the photoprotection-related parameters evaluated under control (Sin150) and stationary phase (Stat) conditions ^1,2^.

	Sin150	Stat	Sin150	Stat	Sin150	Stat	Sin150	Stat	Sin150	Stat	Sin150	Stat
	ABTS		AsA		Phenolics		Flavonoids		Dd		Dt	
AsA	0.83	0.83										
Phenolics	0.87	0.95	0.81	0.80								
Flavonoids	0.84	n.s	0.60	n.s	0.76	n.s						
Dd	0.64	n.s	0.53	n.s	0.58	n.s	0.62	n.s				
Dt	0.60	0.70	0.63	n.s	0.61	0.62	0.66	n.s	n.s	n.s		
β-car	0.54	n.s	0.47	n.s	0.51	n.s	0.54	n.s	n.s	n.s	0.84	−0.58
Light	0.75	n.s	0.68	n.s	0.72	n.s	0.75	0.61	n.s	n.s	0.57	n.s
NPQ	n.s	n.s	n.s	n.s	n.s	n.s	n.s	−0.67	n.s	n.s	n.s	n.s

^1^ Abbreviations and units used for the correlation are as follows: Ascorbic acid (AsA in fg/cell); Phenolics (in fg GAEq/cell); Flavonoids (in fg QEq/cell); ABTS test (in fg AEQ/cell); Diatoxanthin (Dt in fg/cell); Diadinoxanthin (Dd in fg/cell); β-carotene (β-car in fg/cell); Light (in μmol photons m^−2^ s^−1^). ^2^ n.s. = non-significant (*p*-value > 0.01).

**Table 5:** Spearman correlation matrix between the antioxidant capacity, antioxidant molecules, and the photoprotection-related parameters evaluated under light conditions ^1^.

	Sin600	Quad600	Quad300	Sin600	Quad600	Quad300	Sin600	Quad600	Quad300	Sin600	Quad600	Quad300	Sin600	Quad600	Quad300	Sin600	Quad600	Quad300
	ABTS			AsA			Phenolics			Flavonoids				Dd			Dt	
AsA	n.s	n.s	0.82															
Phenolics	0.59	n.s	n.s	n.s	0.67	n.s												
Flavonoids	n.s	0.74	n.s	n.s	n.s	n.s	0.64	n.s	n.s									
Dd	n.s	n.s	n.s	n.s	n.s	n.s	n.s	n.s	n.s	n.s	n.s	n.s						
Dt	0.56	n.s	n.s	n.s	n.s	n.s	n.s	0.81	n.s	n.s	n.s	n.s	n.s	n.s	n.s			
β-car	0.76	n.s	n.s	n.s	n.s	n.s	n.s	n.s	n.s	n.s	n.s	n.s	0.71	0.81	0.89	0.56	n.s	n.s
Light	0.61	n.s	n.s	0.79	n.s	n.s	n.s	0.63	n.s	0.61	n.s	n.s	n.s	n.s	n.s	0.76	0.76	n.s
NPQ	n.s	n.s	n.s	n.s	n.s	0.93	n.s	n.s	n.s	n.s	n.s	n.s	n.s	0.74	n.s	n.s	n.s	n.s

^1^ Abbreviations and measurement units as in Table 4.

**Table 6:** Spearman correlation matrix between the antioxidant capacity, antioxidant molecules, and the photoprotection-related parameters evaluated under low light and dark conditions ^1^.

	Dark	Con10	Sin10	Dark	Con10	Sin10	Dark	Con10	Sin10	Dark	Con10	Sin10	Dark	Con10	Sin10	Dark	Con10	Sin10
	ABTS			AsA			Phenolics			Flavonoids			Dd			Dt		
AsA	n.s	n.s	n.s															
Phenolics	n.s	n.s	0.82	n.s	n.s	n.s												
Flavonoids	n.s	0.90	n.s	n.s	n.s	n.s	n.s	0.90	n.s									
Dd	n.s	n.s	n.s	n.s	n.s	n.s	n.s	n.s	n.s	n.s	n.s	n.s						
Dt	0.61	n.s	n.s	n.s	n.s	n.s	n.s	n.s	n.s	n.s	n.s	n.s	0.80	n.s	n.s			
β-car	0.70	n.s	n.s	n.s	0.82	n.s	n.s	n.s	n.s	n.s	n.s	0.81	0.56	0.82	n.s	0.68	n.s	n.s
Light	n.s	0.87	n.s	n.s	n.s	n.s	n.s	0.87	n.s	n.s	0.87	n.s	n.s	n.s	n.s	n.s	n.s	n.s
NPQ	n.s	n.s	–0.96	n.s	n.s	n.s	n.s	n.s	–0.89	n.s	n.s	n.s	n.s	n.s	n.s	–0.78	n.s	n.s

^1^ Abbreviations and measurement units as in Table 4.

## References

[1] Foyer C.H., Shigeoka S. (2011). Understanding Oxidative Stress and Antioxidant Functions to Enhance Photosynthesis. Plant Physiol..

[2] Mittler R. (2002). Oxidative stress, antioxidants and stress tolerance. Trends Plant Sci..

[3] Foyer C.H., Noctor G. (2005). Oxidant and antioxidant signalling in plants: A re-evaluation of the concept of oxidative stress in a physiological context. Plant Cell Environ..

[4] Foyer C.H., Noctor G. (2005). Redox homeostasis and antioxidant signaling: A metabolic interface between stress perception and physiological responses. Plant Cell Environ..

[5] Snoeijs P., Sylvander P., Häubner N., Abele D., Vázquez-Medina J.P., Zenteno-Savín T. (2012). Oxidative stress in aquatic primary producers as a driving force for ecosystem responses to large-scale environmental changes. Oxidative Stress in Aquatic Ecosystems.

[6] Smirnoff N., Wheeler G.L. (2000). Ascorbic acid in plants: Biosynthesis and function. Crit. Rev. Biochem. Mol. Biol..

[7] Stahl W., Sies H. (2003). Antioxidant activity of carotenoids. Mol. Asp. Med..

[8] Boon C.S., McClements D.J., Weiss J., Decker E.A. (2010). Factors influencing the chemical stability of carotenoids in foods. Crit. Rev. Food Sci. Nutr..

[9] Kuczynska P., Jemiola-Rzeminska M., Strzalka K. (2015). Photosynthetic pigments in diatoms. Mar. Drugs.

[10] Marí M., Morales A., Colell A., García-Ruiz C., Fernández-Checa J.C. (2009). Mitochondrial glutathione, a key survival antioxidant. Antioxid Redox Signal..

[11] Krieger-Liszkay A. (2006). Singlet oxygen production in photosynthesis. J. Exp. Bot..

[12] Cheynier V., Comte G., Davies K.M., Lattanzio V., Martens S. (2013). Plant phenolics: Recent advances on their biosynthesis, genetics, and ecophysiology. Plant Physiol. Biochem..

[13] Pietta P.G. (2000). Flavonoids as antioxidants. J. Nat. Prod..

[14] Goiris K., Muylaert K., Voorspoels S., Noten B., De Paepe D., Baart G.J.E., De Cooman L. (2014). Detection of flavonoids in microalgae from different evolutionary lineages. J. Phycol..

[15] Klejdus B., Lojková L., Plaza M., Snóblová M., Sterbová D. (2010). Hyphenated technique for the extraction and determination of isoflavones in algae: Ultrasound-assisted supercritical fluid extraction followed by fast chromatography with tandem mass spectrometry. J. Chromatogr. A.

[16] Xia S., Wang K., Wan L., Li A., Hu Q., Zhang C. (2013). Production, characterization, and antioxidant activity of fucoxanthin from the marine diatom *Odontella aurita*. Mar. Drugs.

[17] Foo S.C., Yusoff F.M., Ismail M., Basri M., Yau S.K., Khong N.M.H., Chan K.W., Ebrahimi M. (2017). Antioxidant capacities of fucoxanthin-producing algae as influenced by their carotenoid and phenolic contents. J. Biotechnol..

[18] Galasso C., Corinaldesi C., Sansone C. (2017). Carotenoids from Marine Organisms: Biological Functions and Industrial Applications. Antioxidants.

[19] Rico M., López A., Santana-Casiano J.M., Gonzàlez A.G., Gonzàlez-Dàvila M. (2013). Variability of the phenolic profile in the diatom *Phaeodactylum tricornutum* growing under copper and iron stress. Limnol. Oceanogr..

[20] Sushanth V.R., Rajashekhar M. (2015). Antioxidant and antimicrobial activities in the four species of marine microalgae isolated from Arabian Sea of Karnataka Coast. Indian J. Geo-Mar. Sci..

[21] Gastineau R., Turcotte F., Pouvreau J.-B., Morançais M., Fleurence J., Windarto E., Prasetiya F., Arsad S., Jaouen P., Babin M. (2014). Marennine, Promising Blue Pigments from a Widespread Haslea Diatom Species Complex. Mar. Drugs.

[22] López A., Rico M., Santana-Casiano J.M., González A.G., González-Dávila M. (2015). Phenolic profile of *Dunaliella tertiolecta* growing under high levels of copper and iron. Environ. Sci. Pollut. Res..

[23] Jerez-Martel I., García-Poza S., Rodríguez-Martel G., Rico M., Afonso-Olivares C., Gómez-Pinchetti J.L. (2017). Phenolic Profile and Antioxidant Activity of Crude Extracts from Microalgae and Cyanobacteria Strains. J. Food Qual..

[24] Smerilli A., Orefice I., Corato F., Gavalás Olea A., Ruban A.V., Brunet C. (2017). Photoprotective and antioxidant responses to light spectrum and intensity variations in the coastal diatom S keletonema marinoi. Environ. Microbiol..

[25] Lavaud J., Rousseau B., Etienne A.-L. (2004). General features of photoprotection by energy dissipation in planktonic diatoms (Bacillariophyceae). J. Phycol..

[26] Ruban A.V., Lavaud J., Rousseau B., Guglielmi G., Horton P., Etienne A.-L. (2004). The super-excess energy dissipation in diatom algae: Comparative analysis with higher plants. Photosynth. Res..

[27] Lavaud J. (2007). Fast regulation of photosynthesis in diatoms: Mechanisms, evolution and ecophysiology. Funct. Plant Sci. Biotechnol..

[28] Derks A., Schaven K., Bruce D. (2015). Diverse mechanisms for photoprotection in photosynthesis. Dynamic regulation of photosystem II excitation in response to rapid environmental change. BBA Bioenerg..

[29] Goss R., Lepetit B. (2015). Biodiversity of NPQ. J. Plant Physiol..

[30] Waring J., Klenell M., Bechtold U., Underwood G.J.C., Baker N.R. (2010). Light-induced responses of oxigen photoreduction, reactive oxigen species production and scavenging in two diatom species. J. Phycol..

[31] Cartaxana P., Domingues N., Cruz S., Jesus B., Laviale M., Serodio J., Marques da Silva J. (2013). Photoinhibition in benthic diatom assemblages under light stress. Aquat. Microb. Ecol..

[32] Brown M.R., Jeffrey S.W., Volkman J.K., Dunstan G. (1997). Nutritional properties of microalgae for mariculture. Aquaculture.

[33] Kourtchenko O., Rajala T., Godhe A. (2018). Growth of a common planktonic diatom quantified using solid medium culturing. Sci. Rep..

[34] Chandrasekaran R., Barra L., Carillo S., Caruso T., Corsaro M.M., Dal Piaz F., Graziani G., Corato F., Pepe D., Manfredonia A. (2014). Light modulation of biomass and macromolecular composition of the diatom *Skeletonema marinoi*. J. Biotechnol..

[35] Balzano S., Sarno D., Kooistra W. (2011). Effects of salinity on the growth rate and morphology of ten *Skeletonema strains*. J. Plankton Res..

[36] Orefice I., Musella M., Smerilli A., Sansone C., Chandrasekaran R., Corato F., Brunet C. (2019). Role of nutrient concentrations and water movement on diatom’s productivity in culture. Sci. Rep..

[37] Orefice I., Chandrasekaran R., Smerilli A., Corato F., Caruso T., Casillo A., Corsaro M.M., Dal Piaz F., Ruban A.V., Brunet C. (2016). Light-induced changes in the photosynthetic physiology and biochemistry in the diatom *Skeletonema marinoi*. Algal Res..

[38] Brunet C., Chandrasekaran R., Barra L., Giovagnetti V., Corato F., Ruban A. (2014). V Spectral radiation dependent photoprotective mechanism in the diatom *Pseudo-nitzschia multistriata*. PLoS ONE.

[39] Brunet C., Johnsen G., Lavaud J., Roy S., Roy S., Llewellyn C.A., Egeland E.S., Johnsen G. (2011). Pigments and photoacclimation processes. Phytoplankton Pigments. Characterization, Chemotaxonomy and Applications in Oceanography.

[40] Bilger W., Rimke S., Schreiber U., Lange O.L. (1989). Inhibition of Energy-Transfer to Photosystem II in Lichens by Dehydration: Different Properties of Reversibility with Green and Blue-green Phycobionts. J. Plant Physiol..

[41] Eilers P.H.C., Peeters J.C.H. (1988). A model for the relationship between light intensity and the rate of photosynthesis in phytoplankton. Ecol. Model..

[42] Running J.A., Severson D.K., Schneider K.J. (2002). Extracellular production of L-ascorbic acid by *Chlorella protothecoides*, *Prototheca* species and mutants of *P. moriformis* during aerobic culturing at low pH. J. Ind. Microbiol. Biotechnol..

[43] Singleton V.L., Rossi J.A. (1965). Colorimetry of total phenolics with phosphomolybdic-phosphotungstic acid reagents. Am. J. Enol. Vitic..

[44] Li H.-B., Cheng K.-W., Wong C.-C., Fan K.-W., Chen F., Jiang Y. (2007). Evaluation of antioxidant capacity and total phenolic content of different fractions of selected microalgae. Food Chem..

[45] Lamaison J.L.C., Carnet A. (1990). Teneurs en Principaux Flavonoides des Fleurs de Crataegus monogyna Jacq et de Crataegus laevigata (Poiret D. C) en Fonction de la Vegetation. Pharm. Acta Helv..

[46] Hammer Ø., Harper D.A.T., Ryan P.D. (2001). PAST: Paleontological Statistics Software Package for education and data analysis. Palaeontol. Electron..

[47] Giovagnetti V., Flori S., Tramontano F., Lavaud J., Brunet C. (2014). The velocity of light intensity increase modulates the photoprotective response in coastal diatoms. PLoS ONE.

[48] Goiris K., Muylaert K., Fraeye I., Foubert I., De Brabanter J., De Cooman L. (2012). Antioxidant potential of microalgae in relation to their phenolic and carotenoid content. J. Appl. Phycol..

[49] Goiris K., Van Colen W., Wilches I., León-Tamariz F., De Cooman L., Muylaert K. (2015). Impact of nutrient stress on antioxidant production in three species of microalgae. Algal Res..

[50] Quideau S., Deffieux D., Douat-Casassus C., Pouysegu L. (2011). Plant polyphenols: Chemical properties, biological activities, and synthesis. Angew. Chem. Int. Ed. Engl..

[51] Hajimahmoodi M., Faramarzi M.A., Mohammadi N., Soltani N., Oveisi M.R., Nafissi-Varcheh N. (2010). Evaluation of antioxidant properties and total phenolic contents of some strains of microalgae. J. Appl. Phycol..

[52] Duval B., Shetty K., Thomas W.H. (2000). Phenolic compounds and antioxidant properties in the snow alga Chlamydomonas nivalis after exposure to UV light. J. Appl. Phycol..

[53] Kováčik J., Klejdus B., Bačkor M. (2010). Physiological Responses of *Scenedesmus quadricauda* (Chlorophyceae) to UV-A and UV-C Light. Photochem. Photobiol..

[54] Lavens P., Sorgeloos P. (1996). FAO Micro-algae. Manual on the Production and Use of Live Food for Aquaculture.

[55] Agati G., Tattini M. (2010). Multiple functional roles of flavonoids in photoprotection. New Phytol..

[56] Saewan N., Jimtaisong A. (2013). Photoprotection of natural flavonoids. J. Appl. Pharm. Sci..

[57] Zhou R., Su W.H., Zhang G.F., Zhang Y.N., Guo X.R. (2016). Relationship between flavonoids and photoprotection in shade-developed *Erigeron breviscapus* transferred to sunlight. Photosynthetica.

[58] Agati G., Brunetti C., Di Ferdinando M., Ferrini F., Pollastri S., Tattini M. (2013). Functional roles of flavonoids in photoprotection: New evidence, lessons from the past. Plant Physiol. Biochem..

[59] Winkel-Shirley B. (2002). Biosynthesis of flavonoids and effects of stress. Curr. Opin. Plant Biol..

[60] Ghasemzadeh A., Jaafar H.Z.E., Rahmat A. (2010). Identification and Concentration of Some Flavonoid Components in Malaysian Young Ginger (*Zingiber officinale* Roscoe) Varieties by a High Performance Liquid Chromatography Method. Molecules.

[61] Chandra S., Khan S., Avula B., Lata H., Yang M.H., ElSohly M.A., Khan I.A. (2014). Assessment of Total Phenolic and Flavonoid Content, Antioxidant Properties, and Yield of Aeroponically and Conventionally Grown Leafy Vegetables and Fruit Crops: A Comparative Study. Evid. Based Complement. Altern. Med..

[62] Kamtekar S., Keer V., Patil V. (2014). Estimation of Phenolic content, Flavonoid content, Antioxidant and Alpha amylase Inhibitory Activity of Marketed Polyherbal Formulation. J. Appl. Pharm. Sci..

[63] Abalde J., Fabregas J., Herrero C. (1991). β-Carotene, vitamin C and vitamin E content of the marine microalga *Dunaliella tertiolecta* cultured with different nitrogen sources. Bioresour. Technol..

[64] Brown M.R., Miller K.A. (1992). The ascorbic acid content of eleven species of microalgae used in mariculture. J. Appl. Phycol..

[65] Massot C., Stevens R., Génard M., Longuenesse J.-J., Gautier H. (2012). Light affects ascorbate content and ascorbate-related gene expression in tomato leaves more than in fruits. Planta.

[66] Wang J., Zhang Z., Huang R. (2013). Regulation of ascorbic acid synthesis in plants. Plant Signal. Behav..

[67] Vaze K.M., Sharma V.K. (2013). On the adaptive significance of circadian clocks for their owners. Chronobiol. Int..

[68] Mierziak J., Kostyn K., Kulma A., Mierziak J., Kostyn K., Kulma A. (2014). Flavonoids as Important Molecules of Plant Interactions with the Environment. Molecules.

[69] Panche A.N., Diwan A.D., Chandra S.R. (2016). Flavonoids: An overview. J. Nutr. Sci..

[70] Treml J., Šmejkal K. (2016). Flavonoids as Potent Scavengers of Hydroxyl Radicals. Compr. Rev. Food Sci. Food Saf..

[71] Saunders J.A., McClure J.W. (1976). The distribution of flavonoids in chloroplasts of twenty five species of vascular plants. Phytochemistry.

[72] Agati G., Azzarello E., Pollastri S., Tattini M. (2012). Flavonoids as antioxidants in plants: Location and functional significance. Plant Sci..

[73] Jakob T., Goss R., Wilhelm C. (2001). Unusual pH-dependence of diadinoxanthin de-epoxidase activation causes chlororespiratory induced accumulation of diatoxanthin in the diatom *Phaeodactylum tricornutum*. J. Plant Physiol..

[74] Cruz S., Goss R., Wilhelm C., Leegood R., Horton P., Jakob T. (2010). Impact of chlororespiration on non-photochemical quenching of chlorophyll fluorescence and on the regulation of the diadinoxanthin cycle in the diatom *Thalassiosira pseudonana*. J. Exp. Bot..

[75] Vidoudez C., Pohnert G. (2012). Comparative metabolomics of the diatom *Skeletonema marinoi* in different growth phases. Metabolomics.

[76] Grover A. (1993). How do senescing leaves lose photosynthetic activity?. Curr. Sci..

[77] Mae T., Thomas H., Gay A.P., Makino A., Hidema J. (1993). Leaf development in *Lolium temulentum*: Photosynthesis and photosynthetic proteins in leaves senescing under different irradiances. Plant Cell Physiol..

